# GATE: Adaptive learning with working memory by information gating in multi-lamellar hippocampal formation

**DOI:** 10.1371/journal.pcbi.1014438

**Published:** 2026-06-29

**Authors:** Yuechen Liu, Zishun Wang, Chen Qiao, Zongben Xu

**Affiliations:** School of Mathematics and Statistics, Xi’an Jiaotong University, Xi’an, China; Indian Institute of Technology Mandi - Kamand Campus: Indian Institute of Technology Mandi, INDIA

## Abstract

Hippocampal formation (HF) supports both the temporary maintenance of task-relevant information and rapid relearning when task structure is preserved. Here we ask what circuit mechanism can link these two functions within a single framework. We propose a model named Generalization and Associative Temporary Encoding (GATE), whose core idea is a self-gating re-entrant EC3–CA1–EC5–EC3 loop. In each lamella, EC3 provides a memory substrate, CA1 selectively reads out the retained information under CA3 gating, and EC5 feeds back to regulate the next EC3 state. Repeating this loop across dorsoventral lamellae yields representational scales that range from local cue-dependent coding to a broader task-related structure. In simple tasks, the single-lamellar model captures selective maintenance and produces place- and splitter-like CA1 activity. In more complex tasks, the multi-lamellar model develops lap, evidence, trace, and other task-relevant representations. Under structure-preserving changes in sensory coding, positional scaffold, or task parameters, the model reuses learned representations and relearns faster. GATE provides a hypothesis-generating computational framework for studying how hippocampal-like circuit motifs may support selective memory gating and structure-preserving relearning.

## Author summary

A central challenge for the brain is to decide what should be kept in mind and what can be ignored. A second challenge is to reuse what has already been learned when a new task preserves part of the old structure. We studied these two problems together in hippocampal formation. Our model, GATE, is built around one main idea: a self-gated re-entrant loop can actively control whether information is written, maintained, read out, or released. We first show that a single loop is sufficient for simple memory-guided behavior and then show that combining the same loop across layers supports more complex task variables and broader representations. Under related task changes, the model relearns faster by preserving the parts of its representation that remain useful. This provides a simple and interpretable computational hypothesis for how hippocampal-like feedback circuits may link temporary memory gating with flexible relearning.

## 1 Introduction

When an agent faces a new task, two demands arise at once. It must decide what information is worth keeping across time, and it must reuse prior knowledge when the new situation preserves part of the old task structure. These demands correspond to working memory (WM) and generalization, yet their interaction remains poorly understood.

WM refers to a short-term, task-dependent mechanism that maintains and manipulates sequential information over time, serving as a substrate for prediction, planning, and flexible decision-making [1]. In this study, we focus on WM’s temporal integration function. We define generalization more narrowly as transfer to a new context that preserves task structure while changing sensory or environmental details [2,3]. In the present study, we use this term in a restricted sense and focus on reuse of learned structure under related task changes, rather than broad generalization across arbitrarily different task families. These definitions highlight the central question of the present work: how can one circuit retain task-relevant information, ignore irrelevant details, and reuse learned structure in a related setting?

The hippocampal formation (HF) is a natural system in which to study this question. HF activity carries trial-specific and task-relevant information in cognitive maps [3–7], and it can also represent maintained variables such as stimulus order [8], accumulated evidence [9], lap number [10], and elapsed delay [11,12]. At the same time, persistent activity in entorhinal cortex (EC) provides a plausible memory substrate [13–15], and the EC3–CA1–EC5–EC3 re-entrant loop provides a candidate circuit for controlling how such information is written, maintained, and read out [16]. Along the dorsoventral axis, hippocampal representations vary in representational scale and behavioral relevance, with dorsal CA1 carrying more local detail and more ventral regions carrying broader, less cue-specific organization [4,17].

Existing models have explained important parts of hippocampal representation learning, including the Tolman-Eichenbaum machine (TEM) [18], Hebbian-RNN [19], clone structured cognitive graph (CSCG) [20], and plasticity-based models [21]. However, these frameworks do not directly address the specific problem studied here: how a biologically grounded circuit can use selective gating to link temporary information maintenance with later structure reuse.

**Fig 1:**
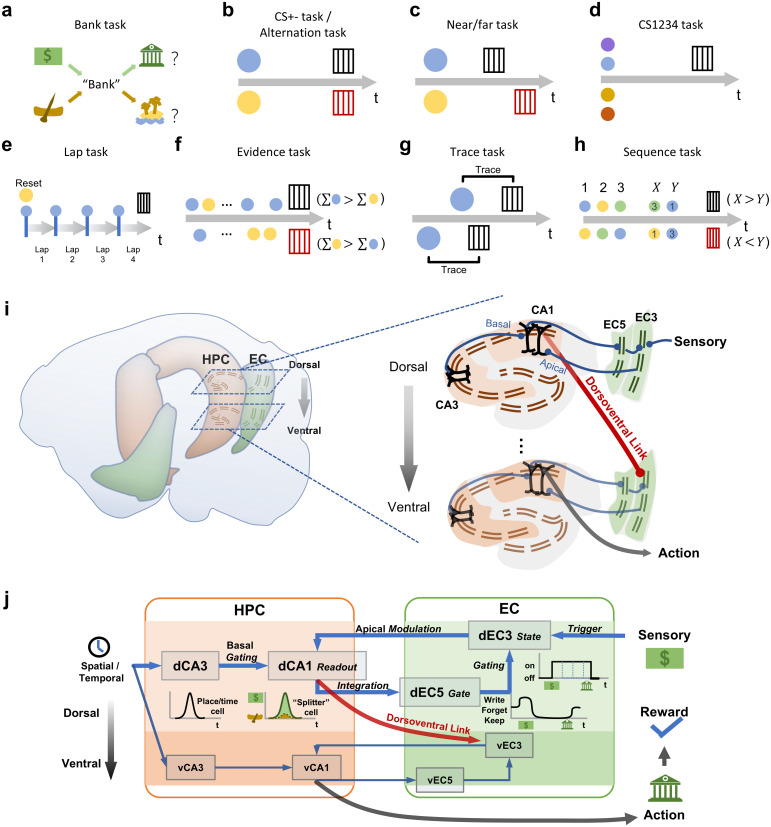
Working-memory tasks, hippocampal formation structure, and the GATE model. **(A)** Schematic example of working memory in language understanding. To determine whether the word “Bank” refers to financial bank (indicated by cash) or river bank (indicated by rowing boat), one needs to keep the context in mind. **(B–H)** Schematic illustration of task structure. Colored circles denote different sensory cues, and rectangles with vertical gratings mark the reward zones. The agent is rewarded at once in the reward zone if action matches sensory cue or task paradigm. The gray arrows represent the direction of motion along a linear track (or circle maze in the Lap task), from left (start point) to right (end point), which also corresponds to the temporal progression of a trial. **(B)** CS + - task: Two cues are presented (randomly one per trial), each indicating a specific correct choice at the track’s end. **(C)** Near/far task: Similar to CS+ but requires actions at different locations. **(D)** CS1234 task: Two of four cues are actionable; the others are not. **(E)** Lap task: The agent resets, completes four laps, and acts at the end of the fourth lap. The environment remains unchanged across laps. **(F)** Evidence task: The agent identifies which of two cues occurs more frequently in a Poisson sequence. **(G)** Trace task: The agent acts after a fixed delay following a random cue. **(H)** Sequence task: The agent determines which of two repeated cues appeared earlier in a three-cue sequence. **(I)** Schematic HF connectivity. Hippocampus (HPC) and EC form a re-entrant loop: EC3 → CA1 → EC5 → EC3. CA3 and EC3 inputs dominate CA1 basal and apical dendrites, respectively. Adjacent lamellas connect dorsoventrally. Sensory input drives dorsal EC3, and ventral CA1 outputs actions. **(J)** Workflow of GATE in the Bank example. EC3 processes sensory input (e.g., “Cash”) and modulates CA1 readout (e.g., activates after “Cash”, not “Boat”). CA3 gates CA1 timing; EC5 integrates CA1 signals and regulates EC3 memory states (write, retain, erase). Correct predictions (e.g., “Financial Bank”) yield rewards.

To address this problem, we propose a network model named Generalization and Associative Temporary Encoding (GATE) (Fig 1J). The conceptual core of GATE is a self-gating mechanism implemented by the EC3–CA1–EC5–EC3 loop. In each lamella, EC3 provides a memory substrate, CA1 selectively reads out that memory under CA3 gating, and EC5 feeds back to regulate the EC3 state. When this loop is combined along the dorsoventral axis, the model can preserve different aspects of experience at different representational scales. In this sense, the single-lamellar model addresses selective maintenance and readout, whereas the multi-lamellar model addresses progressive abstraction and structure-preserving transfer.

Using this framework, we ask three questions. First, can a minimal re-entrant loop generate selective write, keep, read, or forget dynamics and task-relevant CA1 coding? Second, does repeating the same loop across lamellae support more complex, internally structured tasks? Third, when task structure is preserved but part of the input changes, can the model reuse learned representations and relearn faster? We do not present GATE as a full model of hippocampal physiology; instead, it is a circuit-level abstraction designed to isolate how selective memory gating can support task-relevant readout and later structure reuse. The results below suggest that GATE can address these questions within a interpretable circuit-level abstraction inspired by hippocampal organization.

## 2 Results

### 2.1 EC3 persistent activity as a substrate for selective persistence

We first asked what kind of memory substrate is needed before selective readout and transfer can be considered. For the present model, the substrate should support three separable operations: writing new information, keeping relevant information across a delay, and forgetting information when it becomes irrelevant. We therefore begin with EC3 persistent activity [13] as a minimal substrate for selective persistence in simple maintenance tasks such as the CS+ task [4] and the Near/far task [6].

EC3 persistent activity provides a useful biological motivation for modeling write, maintain, and release-like operations. Some EC3 cue-tuned neurons respond to multiple landmark stimuli with different response strengths, thereby encoding external information [22]. EC3 neurons can also exhibit switch-like persistent activity, with firing rates resembling stochastic on/off states. Although such activity is variable, it can encode task-relevant information, including cue and reward locations, even early in learning [15]. These observations motivate a simple modeling assumption: depending on its input, an EC3 subgroup can enter regimes that favor writing new information, maintaining stored information, or releasing information that is no longer needed.

**Fig 2:**
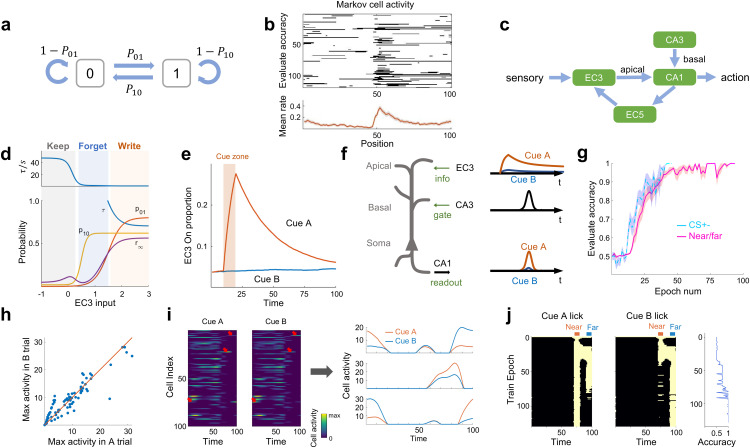
Single-lamellar model learns to maintain information. **(A)** EC3 0/1 state transition. **(B)** Simulated EC3 activity shows stochastic on/off dynamics. Top, activity of a simulated EC3 neuron in response to a pulse input at (45, 55), black indicates on state. Bottom, mean neuron rate across trials (shadow = SEM), similar to [15]. **(C)** Workflow of the single-lamellar model, forming a re-entrant loop. **(D)** EC3 output governed by *P*_01_, *P*_10_, $r_{\infty }$, τ based on EC3 input. Shadowed areas highlight stages of information processing. **(E)** Sensory stimulus generates different EC3 subgroup activity. This subgroup only receives cue A stimulus in the sensory input area (cue zone, shadowed area). Red and blue curves, mean rate of EC3 subgroup. **(F)** CA1 neuron model. Left, semantic CA1 neuron structure. Right, semantic input and output of CA1 neuron. EC3 drives the CA1 apical tuft and CA3 drives CA1 basal dendrites, producing splitter-cell-like activity in the model [29]. **(G)** Training accuracy of Near/far task (shadow = SEM). Performance (y-axis) quantifies per-time-step lick/no-lick accuracy within the task-relevant zones. **(H)** The model develops place-cell-like and splitter-cell-like activity patterns. Red line indicates x = y. **(I)** CA1 activity by trial type. Left, firing rate. Red arrows indicate representative cells shown on right, which show qualitatively similar response profiles to those reported in [29]. **(J)** Agent actions during Near/far task training. Left and middle, raster plots of the “lick” action; yellow pixels denote lick behavior, and black pixels denote no-lick behavior. Right, accuracy curve.

We therefore describe each EC3 subgroup by the fraction of active cells, *r*(*t*). This fraction changes according to the input *I*(*t*) through two transition probabilities: an on-to-off probability *p*_10_(*I*) and an off-to-on probability *p*_01_(*I*) (Fig 2A and 2B). At the population level, *r*(*t*) follows a first-order ODE that converges to a stable fixed point $r_{\infty }$ with time constant τ when *I* is fixed (see Methods). As *I* increases, the model passes through three functional regimes: *keep*, *forget*, and *write* (Fig 2D). The resulting activation and maintenance dynamics are qualitatively similar to EC3 persistent activity reported in [15] (Fig 2B and 2E). Unlike a purely discrete Markov-chain implementation, this population-level formulation can be trained by back-propagation.

### 2.2 A re-entrant loop enables selective readout and self-gating

The EC3 population model provides a substrate for temporary information maintenance. The next question is how stored information should influence behavior only at the appropriate time and place. This requires a selective *read* function [23] and a feedback pathway that can regulate the subsequent state of the memory substrate.

EC3 input to distal or apical CA1 dendrites is strongly attenuated before reaching the soma, and therefore has limited ability to drive somatic spiking on its own. However, distal EC3 input can be amplified when paired with CA3 input onto basal dendrites, a process often described as dendritic gating [24]. In the present model, CA3 provides a positional or temporal scaffold for CA1 readout, whereas EC3 provides contextual information. CA1 therefore combines positional and contextual inputs to form task-dependent representations [25] (Fig 2F). Furthermore, CA1 readout can determine behavioral actions through a linear transformation. The purpose of the single-lamellar model is not to explain every feature of CA1 activity, but to isolate the minimal circuit computation by which retained information becomes selectively readable and behaviorally useful.

EC5 provides a plausible candidate pathway for closing this loop in the present abstraction. EC5 also shows persistent activity but behaves more like a numerical integrator [26], i.e., its firing rate changes only when sufficiently strong excitatory or inhibitory input is given. Combined with the known hippocampal-entorhinal connectivity, this motivates the EC3-CA1-EC5-EC3 re-entrant loop in our model. Specifically, information retained in EC3 is selectively read out by CA1, integrated by EC5, and then fed back to regulate the next EC3 state–namely, whether a subgroup should write new information, maintain the current content, or release it. In this sense, the loop is self-gating: the circuit not only stores information, but also helps determine how that stored information should evolve over time.

This re-entrant architecture defines the single-lamellar model. The model receives sensory input through EC3 and positional or temporal input through CA3, and outputs the current behavioral decision (Fig 2C). It performs well in the CS+ and Near/far tasks (Fig 2G), where performance is quantified as lick/no-lick accuracy within the reward-relevant evaluation zones. Furthermore, the single-lamellar model develops splitter-cell-like task-relevant activity at the cellular level [27–29]. These splitter-like units collectively covered much of the track, with individual units active at distinct spatial locations (Fig 2H and 2I), and provided task-relevant information that could be read out for behavioral choice in the model. Note that fewer CA1 cells exhibit activity in the distal region, consistent with a distributed coding mechanism where the behavioral output is supported by a compact task-relevant subpopulation rather than broad population activation. For visualization, we selected the three CA1 units with the most pronounced cue-dependent differences (Fig 2I). Such strongly modulated cells frequently appear near the end of the track, where behavioral decisions occur. These examples demonstrate that, after learning, different CA1 units develop distinct spatial–contextual tuning and that the same location can evoke different activity patterns across trials, both of which support correct performance in this implementation.

The model also contains CA1 units without clear trial-type tuning. These units may provide additional positional or temporal signals for downstream readout and feedback in the model. In short, the single-lamellar model enables CA1 to selectively read out information held by EC3 populations, thereby guiding the behavior decision, while simultaneously enabling EC5 to integrate and guide the information maintaining process in EC3. Additionally, by analyzing the agent’s behavior (“lick” or “not lick”), we find that the single-lamellar model shows a staged behavioral progression that qualitatively resembles reported rodent behavior (Fig 2J). At the beginning of training, the agent licks broadly across the track. After a short training period, it licks at both reward sites, and eventually develops selective licking at the cue-appropriate reward site [6].

Importantly, localized CA1 fields are not imposed by a fixed CA3 → CA1 mapping. They emerge from random initial weights through training in the full EC3–CA1–EC5–EC3 loop. To test whether this localized tuning depends on a Gaussian CA3 template, we replaced the original Gaussian-like CA3 input with non-Gaussian rectangular-wave position bases; representative CA1 cells still developed localized Gaussian-like responses after learning (S1 Fig). This result suggests that localized and behaviorally useful CA1-like readout is shaped by circuit constraints and task demands in the model. The upstream generation of CA3 spatial fields is not modeled here; CA3 is treated as a simplified scaffold used to isolate the computation of interest.

These results should therefore be read as a proof of principle for the self-gating mechanism, not as a full model of hippocampal representation. The single-lamellar circuit is intentionally minimal: it isolates how the EC3–CA1–EC5–EC3 loop can turn retained information into selective CA1 readout and behaviorally useful gating. This minimal circuit then serves as the building block for the multi-lamellar model.

### 2.3 Combined self-gating across lamellae supports more complex task structure

The single-lamellar model captures key aspects of selective maintenance in simple tasks, but many tasks require more than retaining sensory details. Such tasks also require the circuit to extract internal task variables, such as order [8], accumulated evidence [9], or lap count [10], from similar sensory inputs. This motivates the second design step of GATE: repeating the same self-gating loop across lamellae so that different layers can operate at different representational scales.

**Fig 3:**
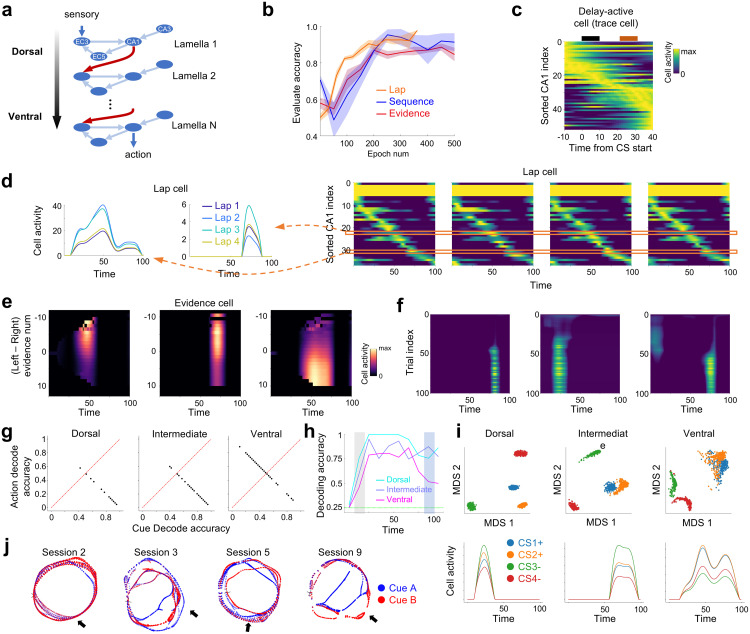
Multi-lamellar model supports simulated working-memory tasks. **(A)** Schematic of the multi-lamellar model. **(B)** Evaluation accuracy in task Lap, Sequence, and Evidence. Shadow area indicates SEM. **(C)** Delay-active cell activity in the Trace task, sorted by maximum activity location, qualitatively similar to [31]. CS, conditional stimulus zone (black bar); US, unconditional stimulus zone (brown bar). **(D)** Cell representation in Lap task. Orange boxes and dashed arrows indicate representative cells on left, similar to [10]. **(E)** Evidence-cell activity in the Evidence task, qualitatively similar to [9] **(F)** Representative cell activity transitions during training, illustrating switching-like patterns similar to [33]. **(G)** Linear decoder accuracy of cue versus action in error evaluation trials across three layers. Retrospective coding was observed across layers, whereas prospective coding was mainly observed in the ventral layer in this model. **(H)** Cue-identity decoding accuracy based on CA1 population activity from dorsal, intermediate, and ventral lamellae in the CS1234 task. Dashed green line indicates chance accuracy. Gray shading, cue zone; blue shading, action zone. Ventral-like CA1 shows reduced cue-identity decoding at the action zone, qualitatively similar to [4]. **(I)** Dorsal (left), intermediate (middle), and ventral (right) CA1 population representations in the CS1234 task. Top, MDS results show that cues with the same task outcome (CS1+ and CS2 + ; CS3- and CS4-) become closer. Bottom, representative neuronal activity in different cue trials. **(J)** Model neural manifold moves toward the task-relevant topology during training in the Near/far task. 20 trials are grouped as one session. Trials start from the black cross and proceed clockwise. Note that the representation gradually decorrelates at the action zone (black arrow), resembling the split-ring-like manifold reported in [6].

To capture externally and internally driven information, we build a multi-lamellar model (Fig 3A). Externally driven information (sensory input) acts as input into the dorsal EC3, and the CA1 readout in ventral lamella guides the actions. In each lamella *i*, ${\text{CA1}}_{i}$ reads out information from ${\text{EC3}}_{i}$. $W_{i}{\text{CA1}}_{i}$, a linear transformation of ${\text{CA1}}_{i}$, is then provides input to ${\text{EC3}}_{i+1}$ in the next lamella, where it is combined with local ${\text{EC5}}_{i+1}$ feedback. Thus, sensory information enters through dorsal EC3, whereas behavioral output is read from the ventral CA1 population.

The multi-lamellar model supports the simulated tasks shown in Fig 3B. In addition to splitter-cell-like and place-cell-like activity, it develops lap-cell-like activity in the Lap task [10], evidence-cell-like activity in the Evidence task [9], and delay-active-like activity in the Trace task [30,31] (Fig 3C, 3D and 3E).

During training, model units also undergo representational transitions resembling tuning changes [6]. Some initially silent units become splitter-cell-like or place-cell-like, whereas some place-cell-like units later become silent (Fig 3F). These switching-like changes are qualitatively similar to rapid representational changes reported in rodent neurons [32,33].

To investigate the presence of retrospective and prospective splitter cells in the model, we adopted the definitions provided by [3] and [5]. We trained the model on a Near/far task, where decisions were made at a single time point near the end of the trajectory, and naturally occurring errors were observed in 41 evaluation trials (Fig 3G). For each CA1 unit, we used its firing-rate profile across the trajectory as the feature vector and trained two separate linear classifiers: one to decode cue identity and the other to decode the chosen action. Cells were classified as retrospective-like if cue decoding was significant and exceeded action decoding, and as prospective-like if action decoding was significant and exceeded cue decoding. The results revealed a predominant presence of retrospective cells in the dorsal and intermediate layers: (i) Dorsal CA1: 39 retrospective cells and 1 prospective cell; (ii) Intermediate CA1: 36 retrospective cells and 0 prospective cells; (iii) Ventral CA1: 7 retrospective cells and 7 prospective cells.

This analysis indicates that, in this model, CA1 activity is primarily retrospective-like, consistent with its working-memory design. The ventral lamella showed a higher proportion of prospective-like cells, which we interpret as a model prediction, rather than as a pattern already established in the experimental literature.

Beyond the single-cell level, we next asked what kind of information is preserved in the population code of each lamella. In Fig 3H, the decoder was trained to predict *cue identity* (CS1–CS4), rather than behavioral output [4]. Thus, this analysis does not quantify splitter-like activity or action readout, but instead measures how much stimulus-specific information is retained in each lamellar population along the trajectory.

The multi-lamellar model shows a representational gradient along the DV axis. In the CS1234 task, dCA1 retains more cue-identity information, whereas vCA1 shows reduced cue-identity decoding at the action zone while preserving information relevant to behavioral outcome (Fig 3H). MDS of CA1 population activity showed a similar lamellar difference (Fig 3I). For all tasks except the Lap task, neuronal activity was reset to zero at the beginning of each trial in the revised implementation. These results are qualitatively consistent with selected aspects of rodent hippocampal population coding reported in [4]. To visualize how neural representations evolve during learning, we also applied uniform manifold approximation and projection (UMAP) to CA1 population activity in the CS1234 task [34]. Each population vector was reduced to a point in 3-D space (Fig 3J). In the model, the neural manifold gradually moved toward a task-relevant topology during learning, qualitatively resembling physiological results in [6].

Taken together, these simulations support the model-level hypothesis that repeated self-gating loops can organize information from more local cue-bound coding toward broader task-related structure, which may facilitate later adaptation to related tasks.

### 2.4 Structure-preserving transfer accelerates relearning

**Fig 4:**
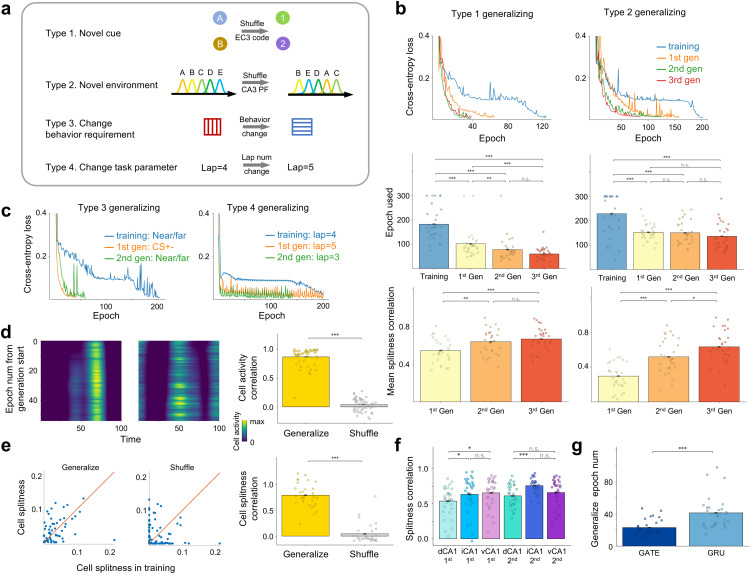
Structure-preserving transfer boosts relearning. **(A)** Scheme of four generalization experiments. PF, place field. **(B)** GATE requires fewer epochs across repeated generalization rounds. Left, Type 1 generalization; right, Type 2 generalization. Top, representative loss curves (loss values above 0.4 are omitted for clarity). Middle, number of epochs required for the classification loss to reach 0.01; training was stopped after 300 epochs if the criterion was not reached. Bottom, splitness-index correlations across generalization rounds. **(C)** Representative loss curve in Type 3 (left) and 4 (right) generalization. **(D)** Place fields remain largely stable during Type 1 generalization. Left, two representative neuronal activity profiles. Right, neuronal activity correlation between training and generalization, or between training and shuffle controls. **(E)** Task-related representations are partially preserved during generalization. Left, scatter plot of splitness during training versus generalization, or training versus shuffle control; red line indicates *x* = *y*. Right, splitness correlation between training and the first or second generalization in dCA1 and intermediate CA1 (iCA1), illustrating lamella-dependent differences in task-related modulation. **(F)** Splitness correlations across training and repeated generalization. Correlations of single-cell splitness were computed between training and the first generalization, and between the first and second generalizations, to assess the stability of task-relevant representations across constrained perturbations. **(G)** Number of epochs required to reach the loss criterion (cross-entropy < 0.1) in the 40-step Near/far benchmark. In all bar plots, *n* = 30; two-sided Mann–Whitney U-tests with Holm–Bonferroni correction for multiple comparisons; *P < 0.05, **P < 0.01, ***P < 0.001, n.s., not significant.

We next asked whether GATE can reuse learned representations when part of the task setting changes but the underlying task structure is preserved. We use “generalization” in this restricted sense: accelerated relearning under constrained perturbations, rather than a full model of hippocampal remapping. To test this, we modified task settings in four ways (Fig 4A): (1) replacing the EC3 input with entirely new sensory coding, corresponding to novel cue types [6]; (2) shuffling all CA3 place fields (or time fields) to perturb the positional scaffold [35]; (3) altering the action requirements, such as replacing a CS + - task with Near/far; and (4) changing task parameters, such as modifying the lap cycle count while preserving the task’s internal logic. Across all conditions, the model relearned faster after prior training (Fig 4B and 4C), consistent with faster adaptation after related cue changes in rodents [4,6].

Under this interpretation, the representation analyses in Fig 4 test which components of the learned code are reused. When EC3 sensory coding is replaced while CA3 positional input is kept fixed, stable CA1 place fields are expected (Fig 4D); the purpose of this analysis is to test whether spatial representations can be reused without catastrophic rewriting while cue-related components are relearned.

When CA3 fields are shuffled, the purpose is complementary: to ask whether task-related modulation can still be partially preserved after the spatial scaffold is changed. We examine a task-relevant representation using a splitness index to quantify each cell’s ability to encode distinct task-related information (Fig 4E). The results reveal a partial stability of splitness, suggesting that task-related modulation can be retained to some extent after positional reshuffling. Notably, intermediate CA1 shows stronger inheritance of these representations than dorsal CA1 in this benchmark, suggesting lamella-dependent differences in representation inheritance (Fig 4F).

For a baseline comparison, we conducted a controlled evaluation between GATE and GRU models trained under identical BP conditions. Both models successfully learned the simplified 30-step Near/far task; however, when the delay was extended to 40 time steps or more, GRUs frequently failed to reach convergence within 100 epochs, consistent with known limitations in gradient propagation across long temporal spans [36]. Fig 4G summarizes the number of epochs required to reach the loss threshold (0.1). Across 30 sessions, GATE required substantially fewer epochs than GRU both during initial learning (mean ± SEM: 41.3±3.5 vs. 66.3±3.7) and generalization (23.3±1.7 vs. 44.3±4.1). Moreover, GRUs showed non-convergence in 63% (19 out of 30) of all 40-step condition sessions, whereas GATE achieved consistent convergence across all runs. These results indicate that, under this controlled benchmark, GATE relearns structurally related but temporally more demanding conditions more efficiently than the GRU baseline.

Accordingly, these results are best interpreted as selective representation inheritance and faster relearning under structure-preserving changes. They do not aim to explain full remapping across arbitrary environments. Within that more specific scope, GATE shows that the same circuit can preserve unaffected components of prior knowledge and update the rest efficiently.

## 3 Discussion

This study addresses a specific problem: how a hippocampal-like circuit can selectively gate task-relevant information over time, and how the same mechanism can support reuse of learned structure in related tasks. We do not aim to model every aspect of hippocampal physiology. Instead, we focus on a circuit-level account of selective maintenance, selective readout, and representational scaling.

Within this scope, the main conceptual contribution of GATE is the self-gating mechanism implemented by the EC3–CA1–EC5–EC3 loop. EC3 provides a memory substrate, CA1 selectively reads it out under CA3 gating, and EC5 feeds back to regulate whether EC3 should write, maintain, or release information at the next step. The ODE and Markov descriptions are not separate innovations. They are two implementations of the same memory substrate at population and discrete levels, respectively.

The single-lamellar model serves as a minimal demonstration of this mechanism. Its purpose is not to reproduce all hippocampal phenomena, nor to claim that CA1 simply copies predefined CA3 tuning. Rather, it isolates how a retained EC3 signal becomes behaviorally useful only when CA3-weighted input opens the readout gate and EC5 completes the feedback loop. In this sense, the single-lamellar model establishes the circuit logic on which the full model is built.

The multi-lamellar extension addresses a second question that the single-lamellar model cannot solve on its own: how the same gating principle can support more complex task variables and broader representational scales. In our model, more dorsal lamellae preserve more local and cue identity-specific structure, whereas more ventral lamellae capture broader outcome-related or task-related organization. This is broadly consistent with the experimental observations reported in [4], but we present this as a computational gradient in the model, rather than as a complete claim about all functions of hippocampus.

The transfer results should be interpreted in the same restricted spirit. Fig 4 does not claim a full model of hippocampal remapping across arbitrary environments. Instead, it shows structure-preserving transfer: when only part of the task setting changes, the circuit can retain unaffected representational components and relearn the changed components more quickly. This interpretation is consistent with reported representational stability and faster relearning after related cue changes [6].

Several experimental findings motivate the memory-maintenance component of the model. Human studies have reported correlations between HF activity during information maintenance and working-memory performance, whereas cue-period HF activity shows weaker relationships [37]. Persistent activity has also been related to working-memory load [38], and entorhinal inputs have been implicated in temporal association memory [39]. In rodents, persistent firing in lateral entorhinal cortex correlates with learning ability [40], and LEC lesions impair associative memory [41]. These findings support the biological plausibility of an entorhinal memory substrate, but they do not directly validate the specific EC3–CA1–EC5–EC3 gating mechanism proposed here.

Another aspect of the GATE model is the differentiation between retrospective and prospective splitter cells, as defined by [5] and [3]. While the model predominantly generates retrospective cells, reflecting working memory encoding, we also observe a small subset of prospective cells. This suggests that the model can contain both retrospective and prospective components. Whether such coexistence reflects biological hippocampal coding remains an empirical question.

Many existing models of hippocampal WM use recurrent networks, attractor dynamics, or synfire-chain activity to maintain information. These mechanisms are complementary to GATE, but they do not directly address how retained information is selectively read out and updated through a hippocampal–entorhinal feedback loop. Lesion studies also suggest that trace association memory cannot be reduced to a simple CA3-only maintenance account: rodents with CA3 lesions can perform some trace tasks, whereas selective CA1 lesions strongly impair trace learning [25,42]. This pattern is compatible with a role for entorhinal–CA1 interactions in temporal association memory, although the present model does not directly simulate lesion effects [43].

In our model, successful task performance requires a CA1-like representation that preserves trial-specific or latent-state-specific information across overlapping locations. In the current implementation, this representation takes the form of splitter-cell activity. We do not claim that classical CA1 splitter cells, as defined experimentally, are uniquely necessary for decision-making *in vivo*. Rather, the model identifies trial-specific hippocampal coding as one computationally useful mechanism for this class of tasks. This interpretation is consistent with the broader experimental literature showing that hippocampal population activity can carry behaviorally relevant prospective information, although such information need not always appear as overt splitter firing at the decision point [28]. Depending on task structure, hippocampal trial-specific activity may instead be expressed during the delay period, maintained as a latent state signal, or be read out by downstream circuits during action selection [3].

GATE is related to temporal-context-style models because both use recent-history signals to support behavior [44]. The key difference is control. In classical TCM accounts, context mainly evolves as a passive trace. In GATE, EC3 persistence is actively regulated by the EC3–CA1–EC5–EC3 loop, so retention, readout, and release are controlled circuit operations. This links a memory trace, a readout gate, and downstream behavior within one mechanism.

We therefore view other timescale-adaptation frameworks, including three-factor rules [45], cascade meta-plasticity [46], and predictive coding [47], as complementary rather than competing accounts. Our specific aim here is narrower: to show how selective memory updating can arise from circuit topology and re-entrant gating.

Many models have addressed cognitive-map formation within a single task and environment, including synfire-chain models [7], TEM [18], Hebbian-RNN [19], CSCG [20], and plasticity-based models [21]. GATE addresses a complementary question: how a hippocampal-inspired gating circuit can link temporary information maintenance with structure-preserving relearning in related tasks.

GATE also differs from standard machine-learning sequence models. Like LSTM and GRU models [48,49], it uses gating to regulate writing, keeping, reading, and forgetting. However, in the controlled benchmark used here, the GRU baseline required more training epochs and showed more frequent non-convergence under longer delays. Transformer architectures provide powerful tools for temporal dependency learning, but their standard formulation is not intended as a biologically constrained model of hippocampal memory gating.

The model suggests several candidate predictions for future experiments: (1) EC3 activity may contain information-maintenance-related components whose persistence depends on task stage; (2) task stage may be decodable from EC5 activity; and (3) behavior-related changes in ventral CA1 representations may precede or differ from dorsal changes [21].

Several limitations follow from the abstraction level of the present model. First, GATE isolates the hippocampal–entorhinal re-entrant loop and does not model broader cortico-subcortical systems that also contribute to learning, action selection, and behavioral flexibility, including prefrontal cortex, basal ganglia, amygdala, thalamic inputs, and other cortical pathways. It also omits other hippocampal regions and pathways, such as dentate gyrus, subiculum, and CA3 recurrent dynamics, which may be important for pattern separation, contextual memory, and lifelong learning. Second, although the model is biologically motivated, it is trained by back-propagation rather than by local synaptic plasticity. The present results therefore do not establish how the proposed self-gating circuit could be learned through biologically plausible learning rules. Third, the model does not include several hippocampal mechanisms, including theta/gamma rhythmic coordination, phase precession, and sharp-wave ripples, all of which may interact with memory maintenance or consolidation. Finally, the transfer experiments are restricted to cases in which the task structure is preserved while selected input components are changed. They should therefore be viewed as tests of accelerated relearning, not as a full account of hippocampal remapping or open-ended generalization. These omissions are intentional simplifications that make the EC3–CA1–EC5–EC3 gating hypothesis tractable, but future work will be needed to test how this circuit motif interacts with plasticity, replay, oscillations, and extra-hippocampal systems *in vivo*.

In summary, GATE shows how a hippocampal-inspired re-entrant circuit can, in principle, link temporary information maintenance, selective readout, and structure-preserving relearning within a single computational framework. The model does not aim to provide a complete account of hippocampal function, but offers a tractable hypothesis for how EC3–CA1–EC5–EC3 feedback may regulate memory gating across task stages. This framework may help guide future experimental and computational studies of hippocampal contributions to working memory, flexible behavior, and biologically inspired learning systems.

## 4 Methods

The model parameters are summarized in Table 1. The following subsections describe the EC3 memory substrate, hippocampal–entorhinal loop architecture, training procedure, task-performance measure, and splitter-cell analyses.

**Table 1:** Model parameters.

Parameter	Value	Units	Description
$N_{cue}$	4 (CS1234 task), 3 (Sequence task), 10 (Trace task), 2 (otherwise)	–	Number of cue types in each task
$N_{EC3}$	100	–	Number of EC3 subgroups (in each lamella)
$N_{CA1}$	100	–	Number of CA1 neurons (in each lamella)
$N_{CA3}$	100	–	Number of CA3 neurons (in each lamella)
$N_{EC5}$	100	–	Number of EC5 neurons (in each lamella)
*dt*	0.1	*dt* (arb.)	Time step
*dx*/*dt*	1	*dv* (arb.)	Velocity of agent in track
*L*	100	*dx* (arb.)	Length of the track
$\Omega_{cue}$	[10,20]	*dx* (arb.)	Cue stimulation zone in track where EC3 subgroups receive cue input
$M_{ij}$	0/1 random matrix with *p*(1)=0.2	–	Cue matrix, which EC3 neurons receive input for each cue type
*c*_01_, *c*_10_	0.001, 0.02	–	Offset of the transition-probability function
*h*_01_, *h*_10_	0.8, 0.6	–	Scale coefficient of the transition-probability function
*m*_01_, *m*_10_	4, 10	–	Stretch coefficient of the transition-probability function
*d*_01_, *d*_10_	1.5, 0.5	–	Shift of the transition-probability function
*D*	5	*dx* (arb.)	Standard deviation of the CA3 place field
*C*_1_, *C*_2_	0.2, 1	–	Offset, scale coefficient of the CA1 activation function
ϵ	0.5	–	Small constant used to prevent division by zero and filter out low-activity neurons
*bs*	32, 128 (Sequence task and Lap task only)	–	Batch size of the training samples
*lr*	0.01	–	Learning rate of the Adam optimizer

### 4.1 EC3 setup and external input

For simplicity, the agent runs with a constant unit velocity through the whole track, such that *x* = *t*.

To keep it clear, we describe the EC3 model at three linked levels. First, EC3 persistent activity is motivated at the single-cell level as stochastic on/off switching. Second, the average behavior of many such cells is written as a population-level ODE, which is the form used for analysis and training. Third, after training, the same population dynamics can be approximated by a discrete Markov realization for simulation. These are not separate models, but three descriptions of the same EC3 memory substrate.

In our model, each cue activates a fixed subset of EC3 subgroups with a short pulse in the cue zone, thereby providing the external signal that can later be written into, maintained within, or released from the EC3 memory substrate. When a task has $N_{cue}$ types of cue, and the *j*-th cue type is deployed in a training trial, the cue stimulates several EC3 neurons as follows:


$$\begin{array}{c} cue_{i}(t)=\chi (t)M_{ij} \end{array}$$ (1)

where *M* is a binary matrix randomly defined before training, so that each cue activates a specific subset of EC3 subgroups; χ is an indicator function that restricts cue input to the cue zone $\Omega_{cue}$:


$$\begin{array}{c} \chi (t)=\left\{ \begin{array}{cc} 1 & \text{if }t\in \Omega_{cue}\\ 0 & \text{otherwise} \end{array}\right. \end{array}$$ (2)

### 4.2 EC3 population model

We model EC3 as the memory substrate of the loop. At the single-cell level, persistent activity is motivated as stochastic on/off switching: a cell may switch from off to on, or from on to off, depending on its current input. This single-cell picture provides the biological intuition for the model. For training and analysis, however, we use a population-level description in which each EC3 subgroup is represented by the fraction of active cells, denoted by $r_{i}(t)$.

Given ${\text{EC3}}_{input}$ (defined below), the probability of transition from on to off is $p_{10}=\gamma_{10}({\text{EC3}}_{input})$, and the probability of transition from off to on is $p_{01}=\gamma_{01}({\text{EC3}}_{input})$, where $\gamma_{10}$ and $\gamma_{01}$ are nonlinear functions:


$$\begin{array}{c} \gamma_{f}(x)=c_{f}+h_{f}\sigma \left[m_{f}(x-d_{f})\right] \end{array}$$ (3)

in which $\sigma (x)=1/(1+e^{-x})$, and f∈{01,10}.

At the population level, these transition probabilities determine the average activity of each EC3 subgroup.


$$\begin{array}{c} \frac{dr_{i}}{dt}=(1-r_{i})p_{01_{i}}-r_{i}p_{10_{i}} \end{array}$$ (4)

When ${\text{EC3}}_{input}$ is fixed, this ODE converges to a stable point $r_{\infty }=p_{01}/(p_{01}+p_{10})$ with time constant $\tau =1/(p_{01}+p_{10})$. In our interpretation, different combinations of $r_{\infty }$ and τ correspond to three functional regimes of the EC3 substrate: writing new information, maintaining currently stored information, and releasing information that is no longer needed.

After training, the same population dynamics can be approximated by a discrete Markov realization (each subgroup is replaced by multiple on/off units with the same input). We use this only as a simulation-level approximation of the trained dynamics, not as a separate model or conceptual contribution.

### 4.3 Model initialization and inter-trial state transition

At the beginning of training, all neurons are initialized with zero activity. For all tasks except the Lap task, the activity states of EC3, EC5, and CA1 are reset to zero at the beginning of each trial. The only exception is the Lap task, in which information from the previous lap must be retained across successive laps by design. For this reason, cross-trial state continuity is preserved only in the Lap task.

### 4.4 Hippocampus formation network

The hippocampal-entorhinal loop is implemented so that EC3 provides the memory substrate, CA3 provides positional or temporal gating, CA1 performs selective readout, and EC5 feeds back to regulate the subsequent EC3 state. We now specify these components in turn. The output of the *m*-th neuron in CA3 is modeled as a Gaussian function on time *t*:


$$\begin{array}{c} g_{m}(t)=\exp\left[-(t-t_{m}{)}^{2}/D^{2}\right] \end{array}$$ (5)

where *D* is the standard deviation and $t_{m}$ is the center of the place field that covers the whole track. For simplicity, the agent moves at constant speed on a periodic linear track, so that the beginning and end of the track are connected.

The *j*-th CA1 neuron can be described by the following multi-compartment model:


$$\begin{array}{cc} b_{j}(t) & =\text{relu}\left(W_{jm}^{basal}g_{m}(t)\right)\\ a_{j}(t) & =\sigma \left(W_{ji}^{apical}r_{i}(t)-\alpha_{j}\right)\\ s_{j}(t) & =\text{relu}\left(b_{j}(t)*(C_{1}+C_{2}a_{j}(t))-\beta_{j}\right) \end{array}$$ (6)

where $b_{j}$ is the basal potential, $a_{j}$ is the apical potential, $W^{basal}$ and $W^{apical}$ are basal and apical weights, $\alpha_{j}$ and $\beta_{j}$ are learnable inhibitory biases, and *C*_1_ and *C*_2_ are constants. In this formulation, CA3 determines when or where CA1 readout is allowed to occur, whereas EC3 modulates what information is amplified at that moment. When the basal potential is weak, the CA1 output is close to zero; in this sense, CA3 input gates CA1 readout. When $b_{j}$ is sufficiently strong, $a_{j}$ acts as a gain factor [50]: the CA1 output is potentiated when $a_{j}$ is large and depressed when $a_{j}$ is small. These cellular mechanisms allow CA1-like units in the model to learn task-relevant activity patterns, including place-cell-like and splitter-cell-like responses.

### 4.5 EC5 feedback and EC3 state update

EC5 acts as an integrative feedback pathway in the loop. At each step, CA1 output is accumulated in EC5, and the resulting EC5 activity contributes to the next EC3 input. Therefore, EC3 state transitions are not determined by sensory input alone but jointly by current external drive and loop feedback. This is the sense in which the EC3–CA1–EC5–EC3 circuit is self-gating.

The *k*-th EC5 neuron integrates its CA1 input:


$$\begin{array}{c} v_{k}(t)=\text{clip}\left(\int_{0}^{t}\phi (W_{kj}^{CA1\to EC5}s_{j}(x))dx\right) \end{array}$$ (7)

where $W^{CA1\to EC5}$ is the CA1-to-EC5 weight, ϕ is a threshold function that ignores small inputs, and the clip function limits EC5 output to [−1,1]. EC5 output is then transmitted to the EC3 input of the same lamella:


$$\begin{array}{c} EC3_{input_{i}}(t)=W_{ik}^{EC5\to EC3}v_{k}(t)+cue_{i}(t)+W_{ij}^{DV}{\tilde{s}}_{j}(t) \end{array}$$ (8)

where $W^{EC5\to EC3}$ is the EC5-to-EC3 feedback weight, $cue_{i}$ is the sensory input, $W^{DV}$ is the dorsoventral transition weight, and ${\tilde{s}}_{j}$ is the CA1 output in the previous lamella. The second term is only included when the EC3 neuron belongs to the dorsal lamella, while the third term is only included in other lamellae.

### 4.6 Retrospective and prospective splitter cell analysis

To distinguish retrospective-like from prospective-like coding, we analyze evaluation trials from the Near/far task under the single-action-point setting, in which the behavioral decision is read out at one predefined position near the end of the trajectory. For each trial, we record the full CA1 activity trace across the trajectory, together with cue identity, chosen action, and trial correctness. Error trials are not artificially introduced, but are the naturally occurring incorrect trials generated during model evaluation.

The analysis is performed at the single-cell level. For each CA1 neuron, the feature vector for one trial is defined as that neuron’s firing-rate profile across the trajectory. Two separate linear classifiers are then trained using correct trials only: one to decode cue identity and the other to decode action. Both classifiers are subsequently tested on the error trials. This design allows us to dissociate whether a neuron primarily reflects the preceding cue or the forthcoming action when the behavioral output is incorrect.

For each neuron, we obtain cue-decoding accuracy and action-decoding accuracy on the error trials. A neuron is classified as retrospective-like if cue decoding is significant and exceeds action decoding, and as prospective-like if action decoding is significant and exceeds cue decoding. Cells with no significant decoding are labeled neutral, whereas cells with significant decoding of both variables but only a small difference between them are labeled ambiguous.

Chance level was estimated with a permutation test rather than assumed directly. For each neuron and decoding target, the training labels were randomly shuffled $N_{perm}=100$ times, the classifier was retrained, and decoding accuracy on the same error trials was recomputed to form a null distribution. A decoding result was considered significant if it exceeded the permutation-based chance level at p < 0.05, where


$$\begin{array}{c} p=\frac{\sum_{k=1}^{N_{perm}}1\;\left(Acc_{perm,k}^{\left(i\right)}\geq Acc_{obs}^{\left(i\right)}\right)+1}{N_{perm}+1}, \end{array}$$ (9)

$Acc_{obs}^{\left(i\right)}$ denotes the observed decoding accuracy of neuron *i*, and $Acc_{perm,k}^{\left(i\right)}$ denotes the decoding accuracy obtained from the *k*-th shuffled-label permutation.

### 4.7 Agent behavior and task performance

Agent behavior is derived from the CA1 output $\hat{s_{j}}$ in ventral lamella through a policy matrix $W^{action}$:


$$\begin{array}{c} q_{n}(t)=W_{nj}^{action}\hat{s_{j}}(t) \end{array}$$ (10)

where *n* indexes possible actions, and the action with the larger *q* value is selected. In our tasks, the agent determines whether to lick the feeding tube at each time step [4], forming a binary classification problem. When reward is available at the feeding tube, the agent should lick; otherwise, it should withhold licking. Given these labels, the model is trained by back-propagation using a weighted cross-entropy loss and the Adam optimizer with learning rate *lr*. The data are batch-normalized to accelerate training with batch size *bs*. During generalization, $W^{action}$ is reset while other weights are retained.

In all of the tasks, task performance quantifies whether the agent correctly performs licking behavior at the appropriate decision step. Specifically, the task is formulated as a binary classification problem, where the model predicts whether a lick should occur (“lick” = 1, “no lick” = 0). For example, in the near/far task, licking should occur only in the corresponding reward zone depending on the cue [6]. Performance was computed as the proportion of correctly predicted lick or no-lick states within the evaluation zone(s) rather than the whole track, providing a model-level analogue of behavioral lick accuracy. In tasks such as the near/far task, this definition implies a chance level of 50%, because one evaluation zone is labeled as “lick” and the other as “no lick” for a given cue. Hence, always licking (or always withholding licking) yields approximately 50% correct responses.

### 4.8 Splitness index

The splitness index $S_{j}$ of the *j*-th CA1 cell is defined as follows:


$$\begin{array}{c} S_{j}=\frac{{\text{std}}_{l}\left(max_{t}\left[{\bar{s}}_{j}^{l}(t)\right]\right)}{{\text{mean}}_{l}\left(max_{t}\left[{\bar{s}}_{j}^{l}(t)\right]\right)+\epsilon } \end{array}$$ (11)

where ${\bar{s}}_{j}^{l}$ is the mean neuronal output in trials with cue type *l*, and ϵ is a sma*ll* constant used to prevent division by zero and filter out low-activity neurons.

## Supporting information

S1 Fig Localized CA1 tuning emerges without assuming a Gaussian CA3 template. This supplementary figure shows that CA1 neurons can develop localized Gaussian-like tuning even when the upstream CA3 inputs are non-Gaussian rectangular-wave basis functions, supporting that the observed CA1 tuning is learned rather than trivially inherited from the CA3 input template.
(PDF)
