# Supplementary material for: GATE: Adaptive learning with working memory by information gating in multi-lamellar hippocampal formation
Source: PLoS Comput Biol. 2026 Jun 29;22(6):e1014438. doi: 10.1371/journal.pcbi.1014438 (PMC13336475; doi:10.1371/journal.pcbi.1014438)
Supplement: S1 Fig — This supplementary figure shows that CA1 neurons can develop localized Gaussian-like tuning even when the upstream CA3 inputs are non-Gaussian rectangular-wave basis functions, supporting that the observed CA1 tuning is learned rather than trivially inherited from the CA3 input template. (PDF) [file pcbi.1014438.s001.pdf]

Supplementary Information for "GATE: Adaptive Learning with Working Memory by Information Gating in Multi-lamellar Hippocampal Formation"

Supplementary Figure S1

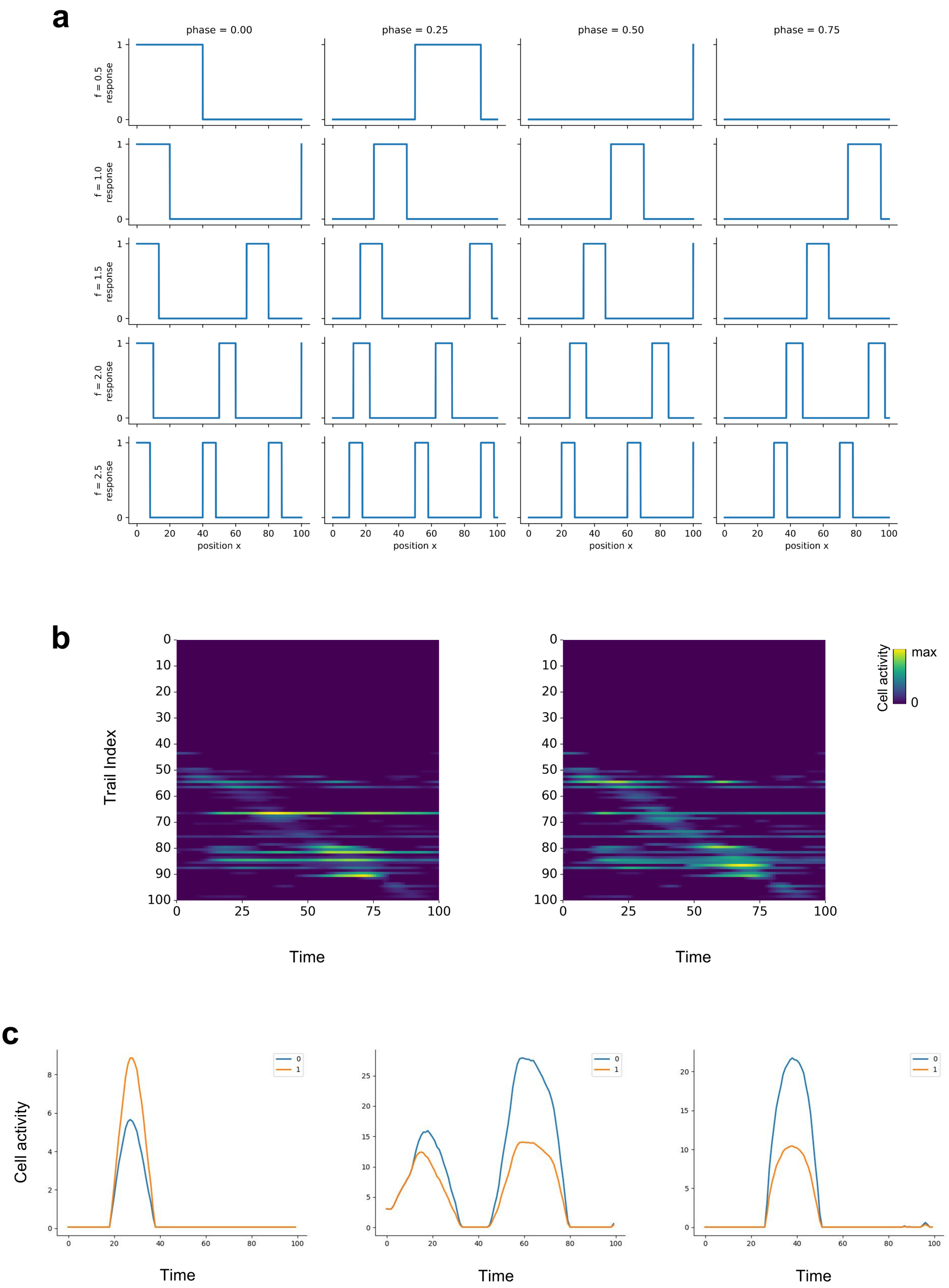

Supplementary Figure S1. Localized CA1 tuning emerges without assuming a Gaussian CA3 template.

(a) Representative non-Gaussian CA3 position scaffold. Here, the CA3 inputs are rectangular-wave position basis functions spanning different frequencies and phases. Each row corresponds to one frequency, and each column to one representative phase. This manipulation preserves positional coverage while removing the assumption that CA3 itself has Gaussian tuning.

(b) Population activity of CA1 neurons after training under the non-Gaussian CA3 scaffold. The heat maps are shown separately for cue A and cue B. Cells are sorted by the position of maximal firing. Despite the rectangular-wave upstream scaffold, CA1 still develops spatially localized activity patterns after learning.

(c) Representative CA1 neurons with localized learned responses. Three example CA1 neurons with distinct tuning profiles are shown, with the two cue conditions overlaid in each panel (blue for cue=A, orange for cue=B). These examples show that localized CA1 tuning is a learned readout shaped by task demands and the gating dynamics of the EC3–CA1–EC5–EC3 loop, rather than a trivial copy of the CA3 input profile.

In the single-lamellar model, localized CA1 fields are not imposed by a fixed CA3→CA1 mapping. Instead, the CA3→CA1, EC3→CA1, and CA1→EC5 pathways are randomly initialized and jointly optimized during training. After learning, CA1 neurons form Gaussian-like localized tuning curves even though the upstream CA3 inputs are non-Gaussian rectangular-wave basis functions. This result supports the interpretation that CA3 is treated here as a simplified positional scaffold, whereas the final CA1 tuning emerges from learning in the full EC3–CA1–EC5–EC3 loop and reflects circuit dynamics together with task demands.
